# The effects of the probiotic cocktail on modulation of the NF-kB and JAK/STAT signaling pathways involved in the inflammatory response in bowel disease model

**DOI:** 10.1186/s12865-022-00484-6

**Published:** 2022-03-03

**Authors:** Shadi Aghamohammad, Amin Sepehr, Seyedeh Tina Miri, Saeideh Najafi, Mahdi Rohani, Mohammad R. Pourshafiea

**Affiliations:** 1 Department of Bacteriology, Pasteur Institute of Iran, Tehran, Iran; 2 Department of Microbiology, Science and Research Branch, Islamic Azad University, Tehran, Iran

**Keywords:** *Lactobacillus*, *Bifidobacterium*, JAK/STAT, NF-kB, Anti-inflammation

## Abstract

**Background:**

Probiotics positively affect inflammatory responses, in part, through Janus kinase/signal transduction and activator of transcription (JAK/STAT) and inflammatory signaling pathways. To evaluate the precise effects of probiotics as protective treatment, we aimed to investigate the effectiveness of *Lactobacillus* spp., *Bifidobacterium* spp., and a mixture of these probiotics in modulating the JAK/STAT and inflammatory signaling pathways.

**Methods:**

A quantitative real-time polymerase chain reaction (qPCR) assay was used to analyze the expression of *JAK/STAT* and inflammatory genes (*TIRAP*, *IRAK4, NEMO*, and *RIP*) following HT-29 cell line treatment with sonicated pathogens *Lactobacillus* spp., *Bifidobacterium* spp., and a mixed cocktail. A cytokine assay was also used to evaluate the IL-6 and IL-1β production following the probiotic treatment.

**Results:**

The probiotic cocktail downregulated the *JAK* genes and *TIRAP*, *IRAK4, NEMO*, and *RIP* genes in the NF-kB pathway compared to sonicate pathogen treatment cells. The expression of *STAT* genes was variable following probiotic treatment. The IL-6 and IL-1β production decreased after probiotic treatment.

**Conclusions:**

Our probiotic cocktail showed anti-inflammatory effects on HT-29 cells by modulating JAK/STAT and NF-kB pathways. Therefore, *Lactobacillus* spp. and *Bifidobacterium* spp. probiotics as nutritional supplements may reduce inflammation-associated diseases such as inflammatory bowel disease (IBD).

## Introduction

The gastrointestinal tract (GT) is an ecosystem for critical bacteria, including beneficial bacteria, which have different effects on the immune system, host metabolism, and microbial balance improvement [1]. According to the World Health Organization (WHO) and the Food and Agriculture Organization (FAO), probiotics are beneficial microorganisms with perceptible advantages and limited side effects when used in appropriate amounts and compositions [2]. Different genera, including *Bifidobacteria*, *Lactobacilli*, and other producing Lactic Acid Bacteria (LAB), such as *Lactococci* and *Streptococci*, have been extensively studied. In vivo studies have revealed that the mixture of *Lactobacillus* and *Bifidobacterium* has significant effects on dysbiosis reduction [3] through regulating the genes involved in inflammation [4].

As known, TLR4 and NOD2 are signaling pathway components that recognize bacterial Muramyl Dipeptide (MDP) and Lipopolysaccharide (LPS). Inflammatory cascades are triggered via the activation of genes such as TIR domain-containing adaptor protein (TIRAP), IL-1 receptor-associated kinase (IRAK), receptor-interacting serine/threonine-protein kinase 2 (RIP), and NF-kappa-B essential modulator (NEMO) [5, 6]. On the other hand, the Janus kinase/signal transduction and activator of transcription (JAK/STAT) signaling pathway is a significant component of the innate and adaptive immune systems that mediate cytokines, having a critical role in inflammatory diseases [7]. Different JAK/STAT system components act through association with various cytokines. For instance, STAT1 is associated with IFNγ and IL-12, whereas STAT3 is associated with IL-6 and IL-10 pathways [8]. All JAK/STAT system components play various roles, including enhanced immunity against infections, immune cell differentiation and growth, and anti-inflammatory actions, which are critical to hematopoiesis and immunity [9].

Balancing and modulating the immune system and strain-specific anti-inflammatory capacities are the critical features of probiotics [10, 11]. Our previous studies have shown the positive effects of probiotics on modulating and decreasing inflammation phenotypically; however, different molecular pathways that play significant roles in inflammation and the effects of probiotic strains on each pathway should be comprehensively studied. Identifying such pathways could be essential for evaluating the presumed effects of probiotics.

Since a greater efficacy could be achieved when different probiotic strains are involved in a mixture, four *Lactobacillus* spp. and three *Bifidobacterium* spp. were used in the current study, as previously shown as a definite combination. Using two different species of probiotics, specifically in the cocktail form, and examining their effectiveness before inflammation could be helpful to understand how probiotics play a preventive role in challenging diseases, including IBD. Therefore, we aimed to investigate the effectiveness of *Lactobacillus* spp., *Bifidobacterium* spp., and a mixture of these probiotics in modulating JAK/STAT and regulating inflammatory signaling pathways.

## Results

**Fig. 1:**
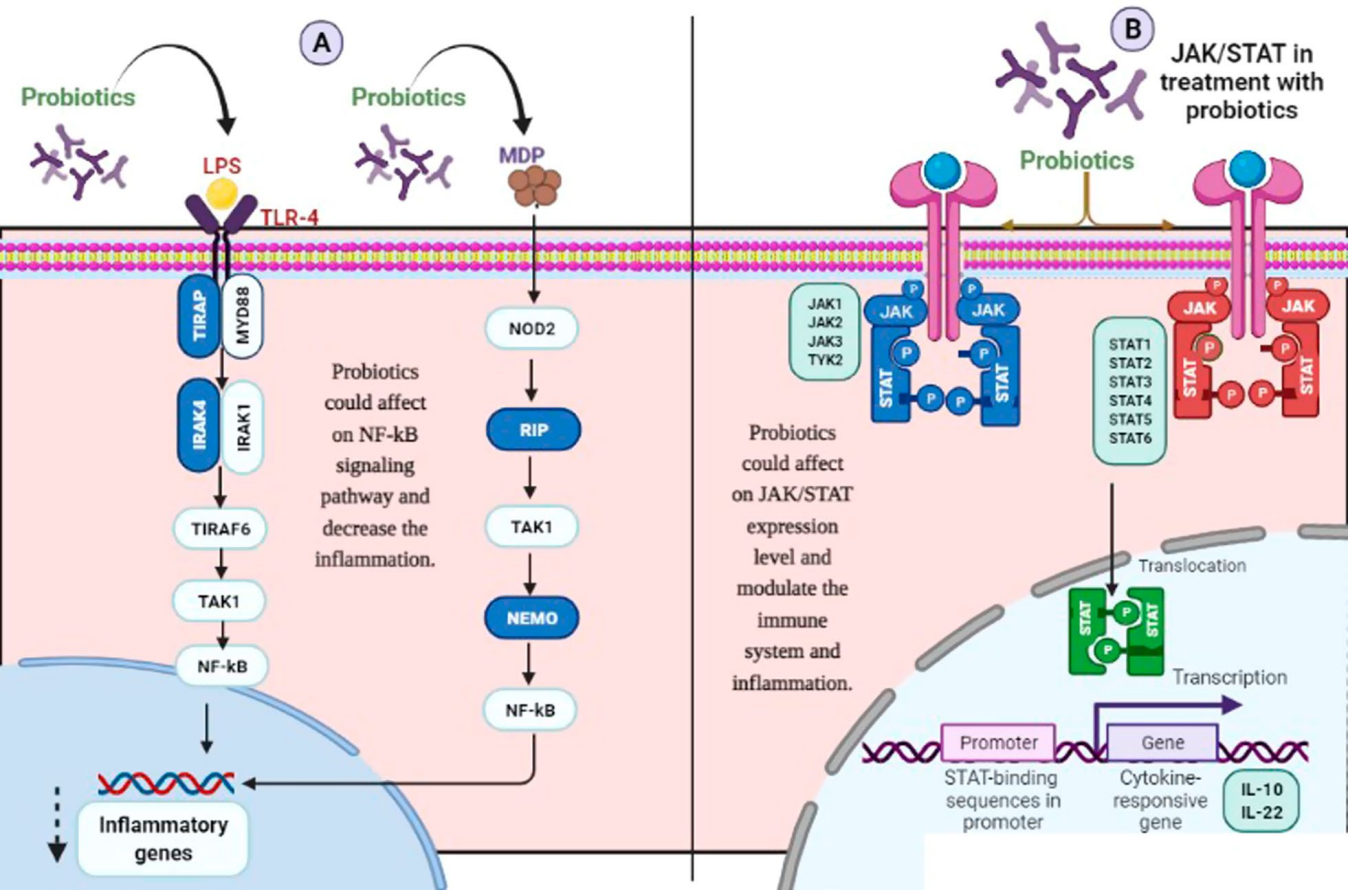
The overal result of probiotic treatments in **a** NF-kB pathway and **b** JAK/STAT

**Fig. 2:**
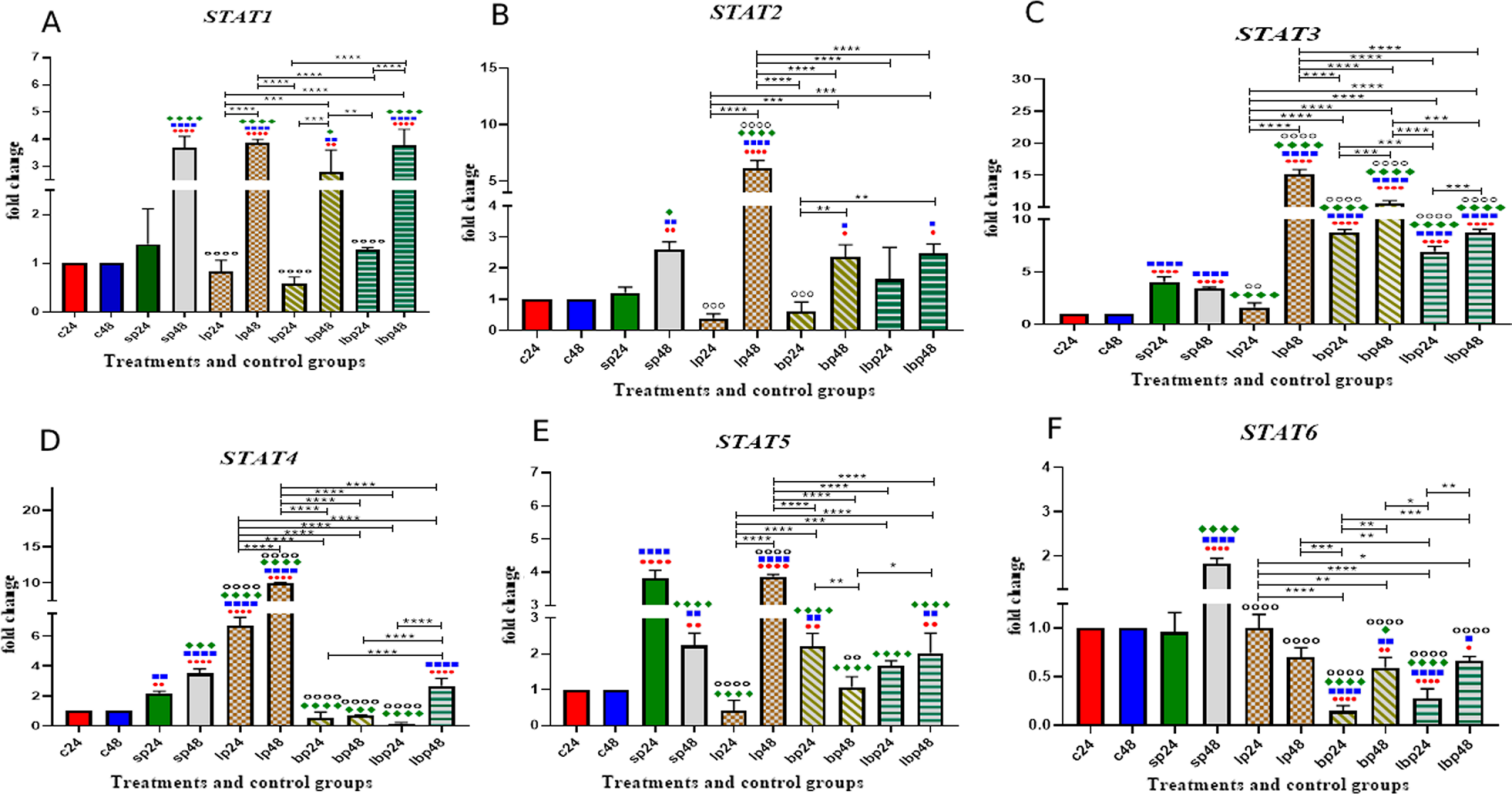
Relative gene expression (mean fold change) of **a** *STAT1*, **b** *STAT2*, **c** *STAT3,* **d** *STAT4,* **e** *STAT5*, and **f** *STAT6* in the different groups of treatments. Data were normalized with *gapdh*. Data were represented as mean SD. The number of 24 and 48 refers to different time orders of HT-29 cell line treatments. *C* control, *P* Pathogen, *LP* first *Lactobacillus* spp. and then pathogen, *BP* first *Bifidobacterium* spp. and then pathogen; LBP, first Lac/Bif and then pathogen. Data were considered as statistically significant when *p* < 0.05 (*p < 0.05, **p < 0.01, ***p < 0.001 and ****p < 0.0001). The red circle indicates the relatedness between C24 with other treatments, Blue Square shows the relatedness between C48 and other treatments, Green diamond indicates the relatedness between P24 with other treatments, and empty circle shows the relatedness between P48 with other treatments. The relatedness between other treatments is shown with brackets

**Fig. 3:**
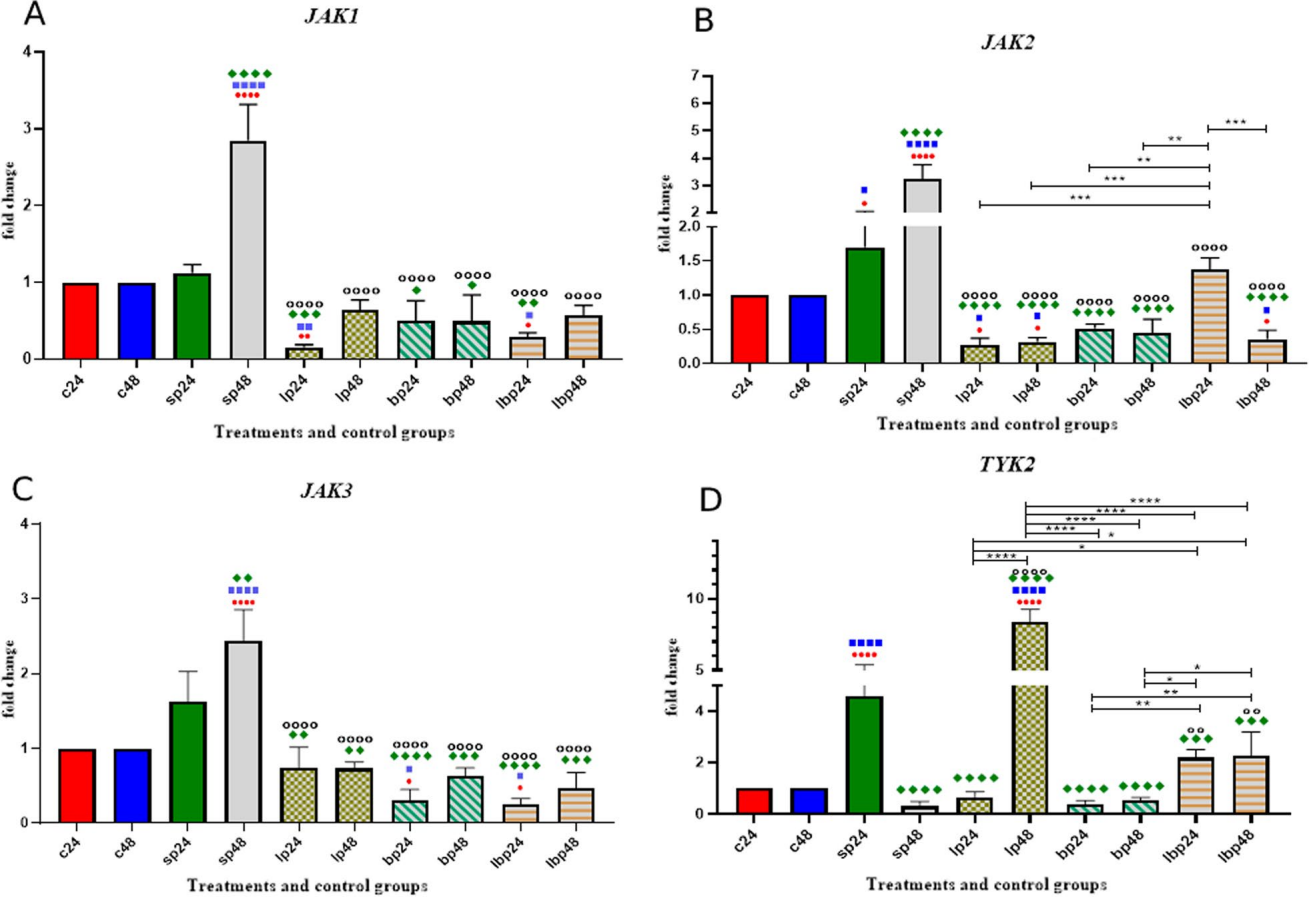
Relative gene expression [mean fold change] of **a** *JAK1*, **b** *JAK2*, **c** *JAK3,* **d**, and *TYK2* in the different groups of treatments. Data were normalized with *gapdh*. Data were represented as mean SD. The number of 24 and 48 refers to different time orders of HT-29 cell line treatments. *C* control, *P* Pathogen, *LP* first *Lactobacillus* spp. and then pathogen, *BP* first *Bifidobacterium* spp. and then pathogen, *LBP* first Lac/Bif and then pathogen. Data were considered as statistically significant when *p* < 0.05 [*p < 0.05, **p < 0.01, ***p < 0.001 and ****p < 0.0001]. The red circle indicates the relatedness between C24 with other treatments, Blue Square shows the relatedness between C48 and other treatments, Green diamond indicates the relatedness between P24 with other treatments, and empty circle shows the relatedness between P48 with other treatments. The relatedness between other treatments is shown with brackets

**Fig. 4:**
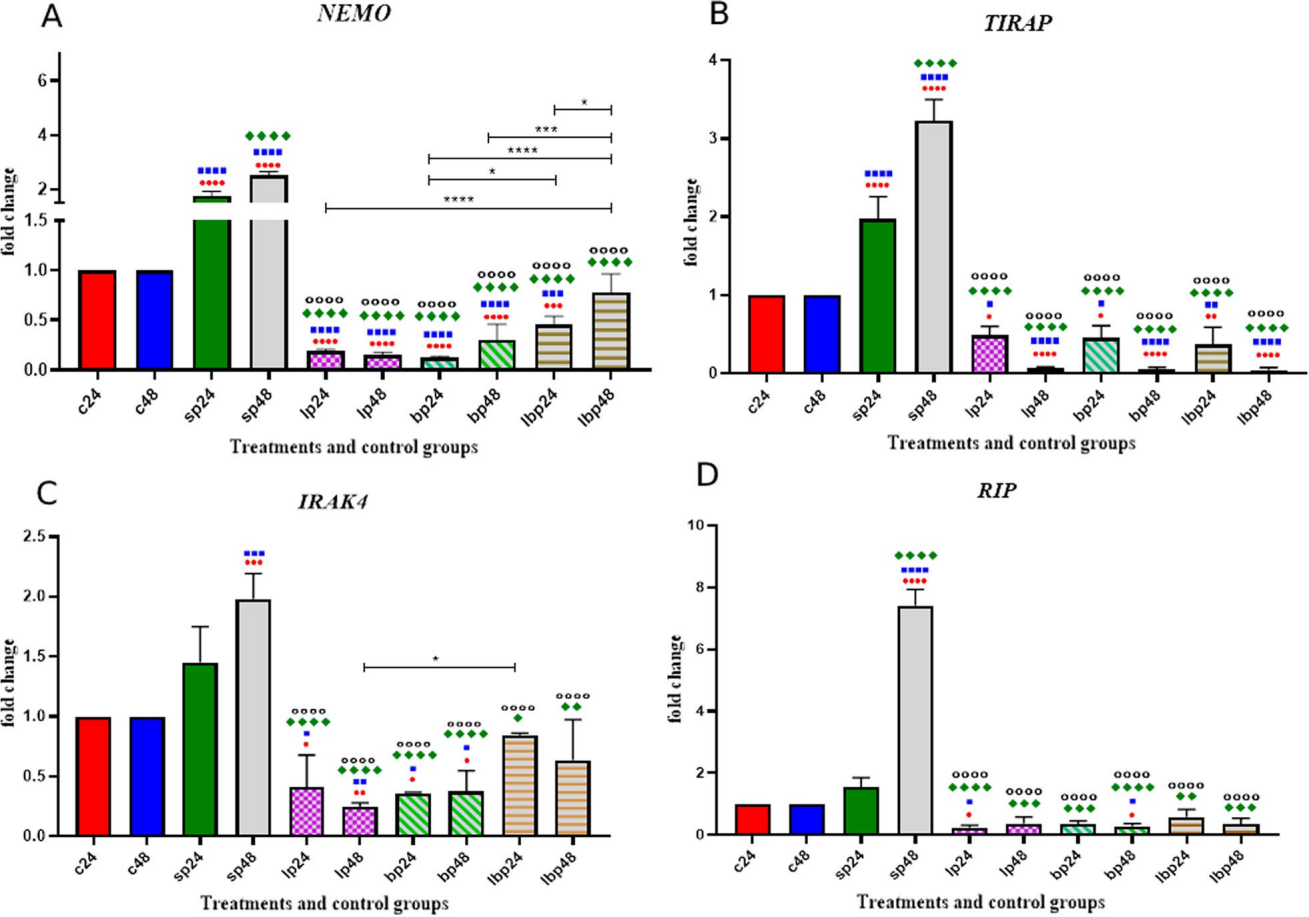
Relative gene expression [mean fold change] of **a** *NEMO*, **b** *TIRAP*, **c** *IRAK,* and **d** *RIP* in the different groups of treatments. Data were normalized with *gapdh*. Data were represented as mean SD. The number of 24 and 48 refers to different time orders of HT-29 cell line treatments. *C* control, *P* Pathogen, *LP* first *Lactobacillus* spp. and then pathogen, *BP* first *Bifidobacterium* spp. and then pathogen; LBP, first Lac/Bif and then pathogen. Data were considered as statistically significant when *p* < 0.05 [*p < 0.05, **p < 0.01, ***p < 0.001 and ****p < 0.0001]. The red circle indicates the relatedness between C24 with other treatments, Blue Square shows the relatedness between C48 and other treatments, Green diamond indicates the relatedness between P24 with other treatments, and empty circle shows the relatedness between P48 with other treatments. The relatedness between other treatments is shown with brackets

The anti-inflammatory effects of the probiotics were previously evaluated phenotypically [1]. A previous in vivo animal model study showed that the length of mice colon was significantly lower in the dextrose sodium sulfate (DSS)-treated group (IBD-induced) than in the normal and probiotic-treated groups. All probiotic strains could significantly (*p* < 0.01) prevent the shortening of the colon [1]. The effectiveness of probiotics in up or downregulation of the inflammatory genes was examined by comparing HT-29 cells treated with probiotics and control cells (not exposed HT-29 cells as negative controls) and HT-29 cells exposed to the sonicated pathogen as a positive control. Also, the statistical differences between all treatments concerning STAT, JAK, and inflammatory genes were examined (Figs. 2, 3 and 4). The outcomes of probiotic treatment concerning the JAK/STAT and NF-kB pathways are summarized in Fig. 1.

### *STAT* gene expression

The data on the *STAT* gene expression are shown in Fig. 2. Comparative analysis of *STAT1* gene expression between sonicated pathogens and negative controls showed that SP-ETEC and SP-ST could significantly increase gene expression after 48 h (*p* < 0.0001). Probiotic treatment (1 h before SP treatment) in the first 24 h significantly decreased the expression level of the *STAT1* gene, while probiotics significantly upregulated *STAT1* gene expression 48 h after the treatments. There was no significant difference between *Lactobacillus* spp. alone *Bifidobacterium* spp. alone, and Lac/Bif in decreasing or increasing the mRNA level of gene expression.

Sonicated pathogens showed a significant increase in *STAT2* expression level after 48 h (*p* < 0.01). *Lactobacillus* spp. and *Bifidobacterium* spp. could significantly decrease the expression level (*p* < 0.001) compared to the positive control (SP48) after 24 h of treatment, while *Lactobacillus* spp. (LP48) had the most significant effects on increasing the expression level (*p* < 0.0001).

Comparative analysis of *STAT3* gene expression between sonicated pathogens and negative controls showed that SP-ETEC and SP-ST could significantly increase the gene expression (*p* < 0.0001). Probiotic treatment (1 h before SP treatment) showed that all the versions could increase the gene expression level, except for treatment with *Lactobacillus* spp. after 24 h (LP24). The most effective treatment for increasing gene expression was *Lactobacillus* spp. after 48 h of treatment (LP48) (*p* < 0.0001). In all probiotic-SP treatments, the increased gene expression level was more significant after 48 h of treatment (*p* < 0.0001 and *p* < 0.001).

Treatments with sonicated pathogens increased the *STAT4* expression level, although the results of probiotic treatments were variable. Using *Lactobacillus* spp*-*SP*.*, after 24 or 48 h, could significantly increase the gene expression (*p* < 0.0001). On the other hand, *Bifidobacterium* spp-SP*.* decreased the *STAT4* expression level after 24 and 48 h of treatments (*p* < 0.0001). Using Lac/Bif-SP could decrease the expression level in the first 24 h of treatment (*p* < 0.0001).

Comparative analysis of *STAT5* gene expression between sonicated pathogens and negative controls showed that SP-ETEC and SP-ST could significantly increase the gene expression, specifically after 24 h. All the probiotic treatments (1 h before SP treatment) decreased the *STAT5* expression level (*p* < 0.0001), except for *Lactobacillus* spp. after 48 h of treatment (LP48), which increased the mRNA level (*p* < 0.0001). *Lactobacillus* spp. had more significant effects on decreasing the gene expression in the first 24 h of treatment (LP24).

Sonicated pathogens significantly increased the expression level of *STAT6* after 48 h of treatment (*p* < 0.0001). All probiotic treatments (1 h before SP treatment) decreased the expression level compared to the positive control (SP48). *Bifidobacterium* spp. and Lac/Bif in the first 24 h of treatment had the most significant effects on decreasing the expression level (Fig. 2).

### *JAK* gene expression

The data on *JAK* expression are shown in Fig. 3. The results of the *JAK* expression level showed homogenous outcomes. For both *JAK1* and *JAK3*, sonicated pathogen treatment could increase the expression level after 24 h of treatment (*p* < 0.0001). All probiotic treatments decreased the mRNA level of *JAK1* and *JAK3* gene expression. There was no significant difference between probiotic treatments.

Comparative analysis of *JAK2* gene expression indicated that sonicated pathogens increased the gene expression. All probiotic treatments (1 h before SP treatment) decreased the mRNA level of gene expression. However, Lac/Bif treatment had the lowest effect on decreasing gene expression.

The *TYK2* expression level increased after using SP in the first 24 h of treatment (*p* < 0.0001). All the probiotic treatments (1 h before SP treatment), except for *Lactobacillus* spp. after 48 h of treatment (LP48), could decrease the gene expression, and LP24 along with *Bifidobacterium* spp*.* had the most significant effect (Fig. 3).

### Inflammatory genes expression

The data on inflammatory gene expression are shown in Fig. 4. The inflammatory genes including *TIRAP, IRAK4, RIP,* and *NEMO* were upregulated following SP-ETEC and SP-ST treatments. In contrast, probiotic treatments downregulated the inflammatory genes. *Lactobacillus* spp*.* and *Bifidobacterium* spp*.* had more significant effects on decreasing *NEMO* expression levels than Lac/Bif treatments. There was almost no significant difference between probiotic treatments concerning other inflammatory genes (Fig. 4).

### Pro-inflammatory cytokines production

**Fig. 5:**
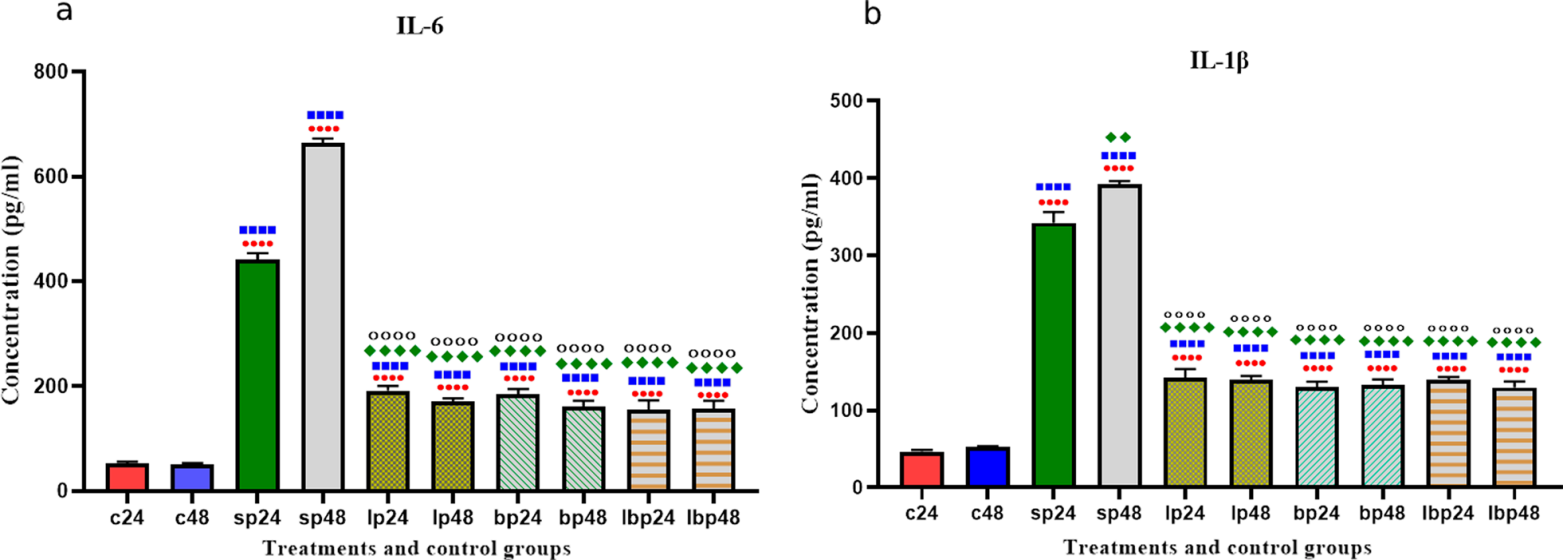
Different levels of concentrations of IL-6 and IL-1β. Data were represented as mean SD. The number of 24 and 48 refers to different time orders of HT-29 cell line treatments. *C* control, *P* Pathogen, *LP* first *Lactobacillus* spp. and then pathogen; *BP* first *Bifidobacterium* spp. and then pathogen, *LBP* first Lac/Bif and then pathogen. Data were considered as statistically significant when *p* < 0.05 [*p < 0.05, **p < 0.01, ***p < 0.001 and ****p < 0.0001]. The red circle indicates the relatedness between C24 with other treatments, Blue Square shows the relatedness between C48 and other treatments, the Green diamond indicates the relatedness between P24 with other treatments, and the empty circle shows the relatedness between P48 with other treatments

The cytokines production significantly increased after SP treatments. However, probiotic treatments (1 h before SP treatment) could significantly decrease cytokine production. No significant difference was seen between *Lactobacillus* spp., *Bifidobacterium* spp*.*, and Lac/Bif at any time after treatment (Fig. 5).

## Discussion

The use of probiotics in immunologically related diseases such as inflammatory bowel disease (IBD) has been studied based on the idea that probiotics can modulate the immune system [12]. The imbalance of pro-inflammatory and anti-inflammatory signaling pathways is the main observation in IBD [13]. It has been shown that colitis and ulcerative colitis diseases, two types of IBD, are associated with different cytokines. Colitis can activate Th1 cells, resulting in the release of Th1-related cytokines such as IFN-γ, while ulcerative colitis is associated with Th2 and the release of IL-5, IL-13, and IL-9. Therefore, finding a way to reduce the signaling molecules producing these cytokines would be potent in managing IBD by decreasing the severity of symptoms [14].

Probiotics are beneficial bacteria with significant roles in preventing and treating IBD [15]. The probiotic strains used in this study were previously shown in our laboratory to have phenotypic anti-inflammatory effects [1]. To understand the genetics of such anti-inflammatory responses, we examined the NF-kB and JAK/STAT signaling pathways following probiotic treatment of the HT-29 cell line. On the other hand, we decided to use probiotics as pre-treatments (1 h before SP treatment) to evaluate the role of probiotics as preventive agents.

The analysis of the NF-kB signaling pathway showed a significant decrease in inflammatory genes following probiotic treatments. Comparing HT-29 cells exposed to SP with our probiotic strains showed reverse effects. On the one hand, SP could increase the expression of the inflammatory genes involved in the NF-kB signaling pathway, whereas our probiotic strains in all forms, alone or mixture, decreased the mRNA level of the studied inflammatory genes. This may explain the molecular mechanism explaining our findings, as reported in a previous study [1], where we showed a significant reduction in IBD-induced inflammatory responses in mice following exposure to these selected probiotics. In support of this notion, the expression level of the *RIP* gene increased following SP treatment. As one of the NOD2 signaling pathway components, *RIP* has a critical role in producing cytokines and causing inflammation in IBD [16]. However, when probiotics were added to HT-29 cells 1 h before SP treatment, more than seven folds decreases were observed in *RIP* gene expression, suggesting the effectiveness of our probiotic strains in controlling the *RIP* gene, which, in turn, could affect the production of the inflammatory cytokines.

Besides, *TIRAP* is one of the critical components of the NF-kB signaling pathway. Different studies reported that the *TIRAP* mutation could reduce cytokine production [17]. Here, *TIRAP* in HT-29 cells decreased more significantly than the other three genes, including *NEMO*, *IRAK4*, and *RIP*. Almost no *TIRAP* gene expression was seen following the treatment of HT-29 cell line with *Lactobacillus* spp., *Bifidobacterium* spp., and Lac/Bif after 48 h of treatment suggesting that our probiotic strains could interfere with cytokine production, mainly if the probiotics would be used longer. Moreover, our preliminary examination of the supernatants of HT-29 cells treated with probiotics showed a significant reduction in IL-6 and IL-1β.

Besides the NF-kB pathway, JAK/STAT plays various roles associated with different types of cytokines, affecting the status of IBD [18]. However, the STATs roles are complicated in inflammatory diseases, including IBD. Taken together, STATs as immunological factors to reduce the IBD severity and improve the inflammatory status. For instance, STAT1 could be upregulated as a defensive reaction so that the inflammation would be limited. Another component of the STAT group, STAT3, is also critical in IBD since it could be associated with some anti-inflammatory cytokines, including IL-10 and IL-22. Although STAT3 acts pro-inflammatory in the adaptive immune system, it appears as a protective agent in the innate immune system. In fact, STAT3 plays a vital role in cellular stress response, apoptosis, and processes involved in wound healing in intestinal epithelial cells. Overall, different components of the STAT family (specifically STAT1, 3, and 5) could affect IBD via activating anti-inflammatory components, promoting integrity maintenance, and regenerating the crypt epithelium [19]. Generally, the JAK/STAT family components, including STAT3 and JAK1, could be affected by different ligands like IL-10 and IL-6 as anti- and pro-inflammatory cytokines; therefore, the JAK/STAT family could play various roles in immune homeostasis [20]. Coskun et al., for example, reported different roles of STAT3 in diverse cell types and noted the complexity and opposite actions of the JAK/STAT family [21]. In the current study, we showed different results in the expression of STAT genes. Some probiotic treatments upregulated, and others downregulated the gene expression level. Both up and downregulation were seen for STAT1, 2, and 4, while STAT3 and STAT5 were approximately upregulated and STAT6 was downregulated. As mentioned earlier, this is because STATs have various roles in immune homeostasis, and probiotics also have immune modulation effects in different diseases like IBD [22]. Therefore, all these variations helped improve the inflammation, in line with other studies. Also, other investigators showed that increased STAT6 could play a significant role in colitis by affecting the IL-13 production [23], whereas a decrease in STAT6 could prevent apoptosis and disruption of cell membrane integrity [24].

Concerning JAK expression, our results showed that probiotics downregulated the expression of JAK genes. Here, our probiotics exhibited anti-inflammatory function similar to JAK inhibitors such as JAKinibs, a drug targeting JAK by reducing JAK expression [25]. Notably, although *Lactobacillus* spp., *Bifidobacterium* spp., and Lac/Bif had different roles in each gene, *Lactobacillus* spp. had the most effects on gene expression. In brief, all tests, including molecular examinations and phenotypic study (cytokine assay), proved decreased inflammation. In general, evaluating probiotics' precise molecular effects on signaling pathways gives a clear view of how probiotics modulate and decrease inflammation. It is critical to improve the life state of people living with IBD and use different treatment methods to reduce the exacerbation of the disease. Medical and surgical treatment of IBD could face some challenges. Finding the simplest way to decrease the symptoms could be significant to IBD patients. As we use probiotics as pre-treatment, the results may suggest that probiotics, in turn, prevent the IBD severity.

## Material and methods

### Bacterial strain, culture medium, and growth conditions

In this study, the in-vitro assay was performed to determine the effects of probiotics on the NF-kB and JAK/STAT signaling pathways. Four *Lactobacillus* spp., including *L. plantarum, L. rhamnosus, L. brevis,* and *L. reuteri*, were isolated from the fecal samples of 53 volunteers of healthy individuals aged 1–36 years [1]. Besides, three *Bifidobacterium* spp., including *B. bifidum, B. longum*, and *B. infantis*, were isolated from breast milk as reported elsewhere [26]. These strains' probiotic and phenotypic characteristics were examined earlier [1, 26].

The bacterial strains were inoculated into MRS broth containing 0.05% l-cysteine and incubated for 20 h at 37 °C. Furthermore, pathogenic bacteria, including enterotoxigenic *Escherichia coli* (ETEC) and *Salmonella typhimurium* (ST), were cultured in Luria–Bertani (LB) broth (Thermo Fisher Scientific, US), followed by sonication to disrupt the cell integrity. To obtain crude sonicated bacterial preparation, we sonicated the heat-killed bacteria (10 rounds, 1 min/round), and the cellular debris was centrifuged (1700*g*, 15 min, 4 °C), resulting in Sonicated Pathogens (SP). All methods were carried out as per the relevant guidelines and regulations. The fecal samples and breast milk were obtained from a previous study, with ethical approval from the Ethics Committee of Pasteur Institute of Iran (IR.PII.REC.1398.060). Signed informed consent forms were obtained from all participants.

### Treatment of HT-29 cells with probiotics

Human colon adenocarcinoma cell line HT-29 was obtained from the Cell Bank of Pasteur Institute of Iran. Then, HT-29 cells were grown in RPMI-1640 (Thermo-Gibco, USA) supplemented with 10% fetal bovine serum (Biochrom, Berlin, Germany) and 1% penicillin–streptomycin (Sigma Aldrich, UK). To perform different treatments, we detached cells by 0.25% Trypsin–EDTA (Gibco, USA), washed them twice with PBS, and counted. The cell suspension was centrifuged, the precipitate was diluted with RPMI-1640, and 2 × 10^5^ cells per well were seeded. For preparing the bacterial suspension, the culture pellet was collected and diluted in RPMI-1640 with 10% FBS without antibiotics to reach an Optical Density (OD) of 0.08 at 600 nm. For the *Lactobacillus*/*Bifidobacterium* mixture (Lac/Bif), equal amounts of the prepared solutions were adjusted at OD of 0.08 at 600 nm for each bacterium, and then, they were mixed.

The HT-29 cells were exposed to different bacteria, either alone or in combination, including sonicated pathogen enterotoxigenic *E. coli* (SP-ETEC), sonicated pathogen *Salmonella typhi* (SP-ST), *Lactobacillus* spp. alone*, Bifidobacterium* spp. alone, and *Lactobacillus*/*Bifidobacterium* mixture (Lac/Bif)*.* Different combinational treatments were done to examine the effects of the probiotic treatments on HT-29 cells upon exposure to SP. The HT-29 cells were treated as follows: *Lactobacillus* spp., *Bifidobacterium* spp., and Lac/Bif were added to the HT-29 cell line. After 1 h, each well was washed twice with PBS for excluding the non-attached bacteria, and then SP-ETEC and SP-ST were added. Then, cell culture was done.

A set of experiments was performed to evaluate probiotics' effects on pathogenic infections. First, according to the determined multiplicity of infection (MOI), probiotic strains were added to each well of six-well culture plates containing HT-29 cells and incubated for 1 h at 37 °C and 5% CO_2_. After incubation, treated cells were washed twice with PBS (pH 7.4). Then, a new RPMI medium without antibiotics containing 10% FBS was added to each well. After 1 h, SP was added and incubated for another 6 h. After incubation, the wells were washed four times with PBS to detach unbound materials. Then, a new RPMI medium without antibiotics containing 10% FBS was added to each well and incubated at 37 °C and 5% CO_2_. These treatments were done in duplicate, and the cell culture was kept for up to 48 h. The MOI was determined as indicated previously [27].

### RT-PCR of inflammatory signaling pathway genes

According to the manufacturer's instructions, the total RNA was extracted using a total RNA extraction kit (Roche, Germany). The quantity and quality of the purified RNA were determined using a NanoDrop1000 UV–Vis Spectrophotometer by measuring absorbance at 260/280 nm. According to the manufacturer's instructions, the cDNA template was synthesized with the cDNA synthesis kit (Yekta Tajhiz, Iran). The online Primer-Bank website (http://pga.mgh.harvard.edu/primerbank) was used to choose the qPCR primers (Table 1). The primers were tested using gradient PCR to get an appropriate annealing temperature. The mRNA of the studied genes was quantified with the ABI Step One Plus detection system (Applied Biosystems, USA) using the SYBR Green master mix (Amplicon Bio, Denmark). All the reactions were performed in duplicate. The formula RQ = 2^−ΔΔCt^ was used to get relative gene expression in the comparative CT method [28]. An appropriate internal control gene, glyceraldehyde 3-phosphate dehydrogenase (*gapdh*), was selected as a housekeeping gene to normalize the data.

**Table 1:** Primer sequences used in this study

Gene	Primer sequence [5′ > 3′]	Primer bank ID	Product size [bp]
STAT1 F	CGGCTGAATTTCGGCACCT	189458859c3	81
STAT1 R	CAGTAACGATGAGAGGACCCT		
STAT2 F	CTGCTAGGCCGATTAACTACCC	291219923c3	87
STAT2 R	TCTGATGCAGGCTTTTTGCTG		
STAT3 F	ACCAGCAGTATAGCCGCTTC	47080104c2	124
STAT3 R	GCCACAATCCGGGCAATCT		
STAT4 F	GCTTAACAGCCTCGATTTCAAGA	345110659c2	91
STAT4 R	GAGCATGGTGTTCATTAACAGGT		
STAT5 F	CGACGGGACCTTCTTGTTG	221316717c3	80
STAT5 R	GTTCCGGGGAGTCAAACTTCC		
STAT6 F	CGAGTAGGGGAGATCCACCTT	296010867c2	92
STAT6 R	GCAGGAGTTTCTATCAAGCTGTG		
JAK1 F	CTTTGCCCTGTATGACGAGAAC	102469033c1	101
JAK1 R	ACCTCATCCGGTAGTGGAGC		
JAK2 F	ATCCACCCAACCATGTCTTCC	223671934c2	121
JAK2 R	ATTCCATGCCGATAGGCTCTG		
JAK3 F	CTGCACGTAGATGGGGTGG	189095272c2	78
JAK3 R	CACGATCAGGTTGGACTTTTCT		
TYK2 F	GAGATGCAAGCCTGATGCTAT	187608614c1	76
TYK2 R	GGTTCCCGAGGATTCATGCC		
RIP2 F	GCCCTTGGTGTAAATTACCTGC	93141034c2	138
RIP2 R	GGACATCATGCGCCACTTT		
NEMO F	AAGAGCCAACTGTGTGAGATG	142381344c1	69
NEMO R	TTCGCCCAGTACGTCCTGA		
TIRAP F	GACCCCTGGTGCAAGTACC	89111123c2	133
TIRAP R	CGACGTAGTACATGAATCGGAG		
IRAK4 F	CTTGGATGGTACTCCACCACT	223671887c3	76
IRAK4 R	AAAATTGATGCCATTAGCTGCAC		

### Cytokine assays

After performing treatments, the cell culture was centrifuged at 6000 rpm, the sediment was discarded, and the supernatant was collected to evaluate pro-inflammatory cytokines, including IL-6 and IL-1β, by ELISA kit (Karmanian Pars Gene, Iran) according to the manufacturer's protocols.

### Statistical analysis

Graphical and statistical analyses were performed using SPSS (ver. 25) and REST 2009 software to compare different groups. Statistical differences between multiple groups, including control (C), pathogen (P), first *Lactobacillus* spp. was given and then pathogen (LP), first *Bifidobacterium* spp. was given and then pathogen (BP), and first Lac/Bif was given and then pathogen (LBP), were determined using ordinary one-way ANOVA. The* p* values < 0.05 were considered statistically significant. The results are presented as standard deviation (SD).

## Data Availability

The datasets generated during and/or analyzed during the current study are available from the corresponding author on reasonable request.
